# Management of Three Rooted Mandibular First Molar with Six Canals: A Case Report

**DOI:** 10.7759/cureus.6280

**Published:** 2019-12-03

**Authors:** Vinoo Subramaniam Ramachandran, Vidhya Shankari, Mensudar Rathakrishnan, Vinodha Chandrasegaran, Karpagavinayagam Kumaraguru

**Affiliations:** 1 Conservative Dentistry and Endodontics, Rathinavel Subramaniam (RVS) Dental College and Hospital, Coimbatore, IND; 2 Conservative Dentistry and Endodontics, Asan Memorial Dental College and Hospital, Chengalpattu, IND; 3 Conservative Dentistry and Endodontics, Sree Balaji Dental College and Hospitals, Chennai, IND; 4 Conservative Dentistry and Endodontics, Peelamedu Samanaidu Govindasamy (PSG) Institute of Medical Sciences and Research, Coimbatore, IND; 5 Conservative Dentistry and Endodontics, Karpaga and Vidhya Dental Hospital, Chennai, IND

**Keywords:** mandibular first molar, additional canal, mid-mesial canal

## Abstract

The objective of dentists is to preserve teeth affected by either caries, trauma, or any other pathological condition. Teeth with deep carious lesions often require endodontic treatment to preserve their form and function. Sound knowledge of root canal anatomy, endodontic pathology, and proper treatment protocol are vital in endodontic treatment success. Currently, there has been an ongoing trend of case reports that highlight the presence of extra canals, which in turn, cautions the clinician to be more prudent. Any missed canal during root canal therapy is the common cause of treatment failure. This case report emphasizes the fact that proper understanding of pulp anatomy combined with the use of modern diagnostic aids and proper treatment protocol is essential for treatment success. This case report describes successful management of three rooted mandibular first molar with extra canals.

## Introduction

The success of endodontic therapy depends on the thorough elimination of infected pulp tissue, microorganisms, and complete three-dimensional sealing of the root canal space. Managing aberrant canals can be difficult, but inability or failure to identify extra canals can lead to treatment failure due to incomplete debridement of the root canal system. The clinician should thus have a thorough knowledge of not only the normal anatomy of the root canal system but possible root canal variations for successful endodontic treatment. Vertucci proposed a standardized method for classifying root canal anatomical variations and divided canal configuration into eight different types based on number of canals from orifices to apex [1]. Weine introduced a clinically significant classification (Type I to Type IV) of the root canal anatomy [2], and new additional canal types were also reported by Sert and Bayirli [3]. The earliest permanent posterior tooth to erupt, mandibular first molar, is most frequently involved in endodontic procedures. Mandibular first molar typically has two roots with the most prevalent root canal system configuration found in mesial and distal roots being Vertucci classification Type IV (two canals run separately from orifice to apex) and Type I (one canal runs from orifice to apex), respectively, but it is common to find other complexities with regard to number of root and root canals. This case report describes successful identification and management of three rooted mandibular first molar with six canals. Three canals were present in mesial root revealing Type XV Sert and Bayirli canal configuration, and the two distal roots had Type II Vertucci canal configuration in one distal root and Type I Vertucci canal configuration in another.

## Case presentation

**Figure 1:**
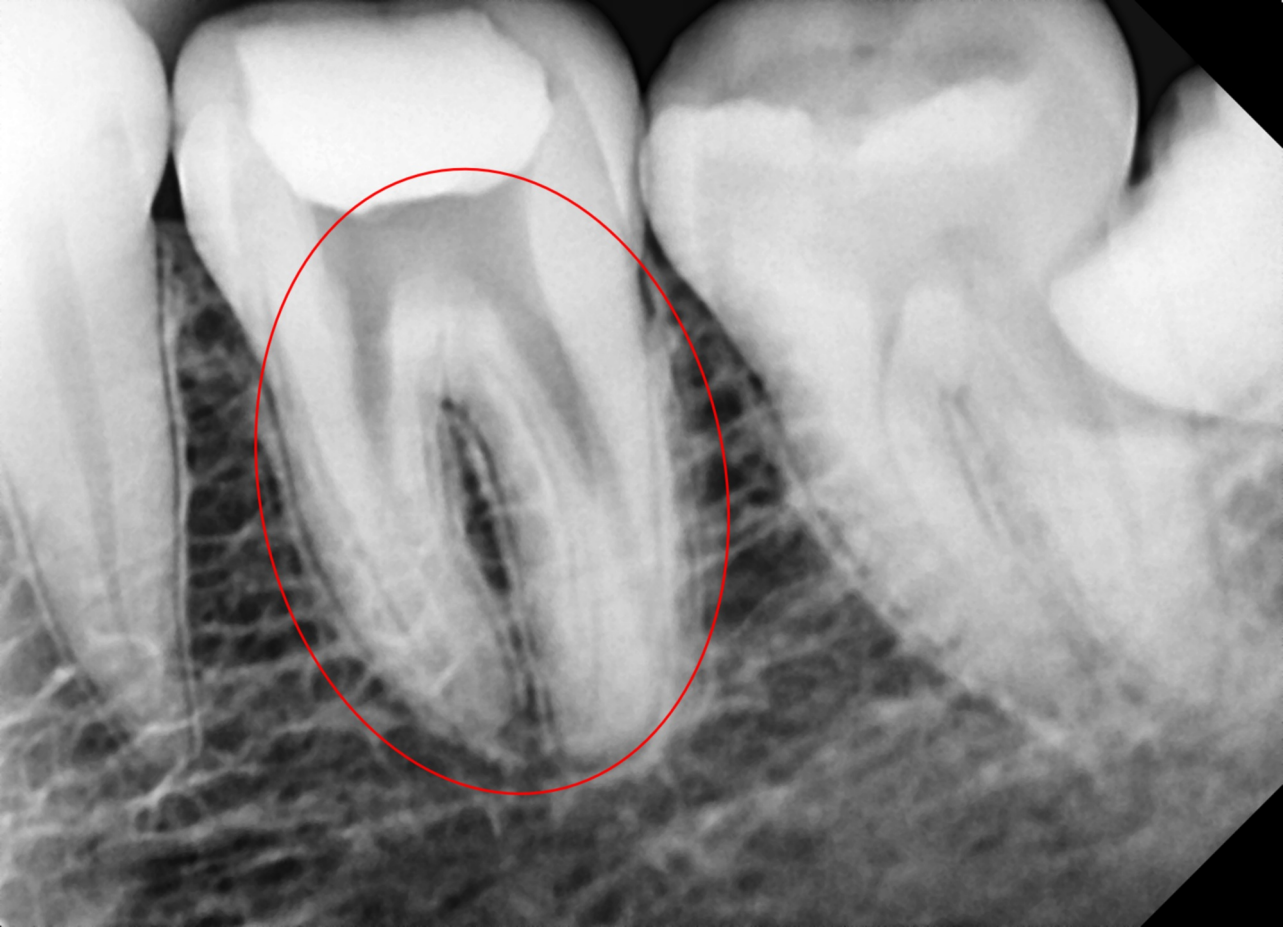
Baseline digital radiograph

A 35-year-old male patient of South Indian origin reported with continuous throbbing pain in a lower left back tooth. The patient was referred by a general dentist who had performed emergency access opening to relieve acute pain in the left mandibular first molar, which was diagnosed with acute irreversible pulpitis one week prior. The patient had a non-contributory medical history. Clinical examination revealed that the left lower first molar (tooth # 36) had access opening done and restored with temporary cement, and the tooth was mildly tender on percussion. Tooth mobility and periodontal probing around the tooth was within physiologic limits. A thorough inspection and interpretation of the baseline intra-oral peri-apical radiograph (Figure 1) was made at the time when the patient presented. The radiograph revealed an unclear view of the canal space as well as variation in the outline of the distal root contour, suggesting possibility of complex root anatomy. Based on history, clinical and radiographic examination, plan for completion of root canal treatment was decided.

The tooth was anesthetized with an inferior alveolar nerve block using 2% lignocaine with 1:80,000 epinephrine (Lignox 2%, Indoco Remedies, Ltd.). Under rubber dam isolation, the temporary restoration was removed, and the pulpal floor was carefully examined with magnification loupe (3.5 x), and three canals (mesiobuccal, mesiolingual, distal canal) were easily identified.

**Figure 2:**
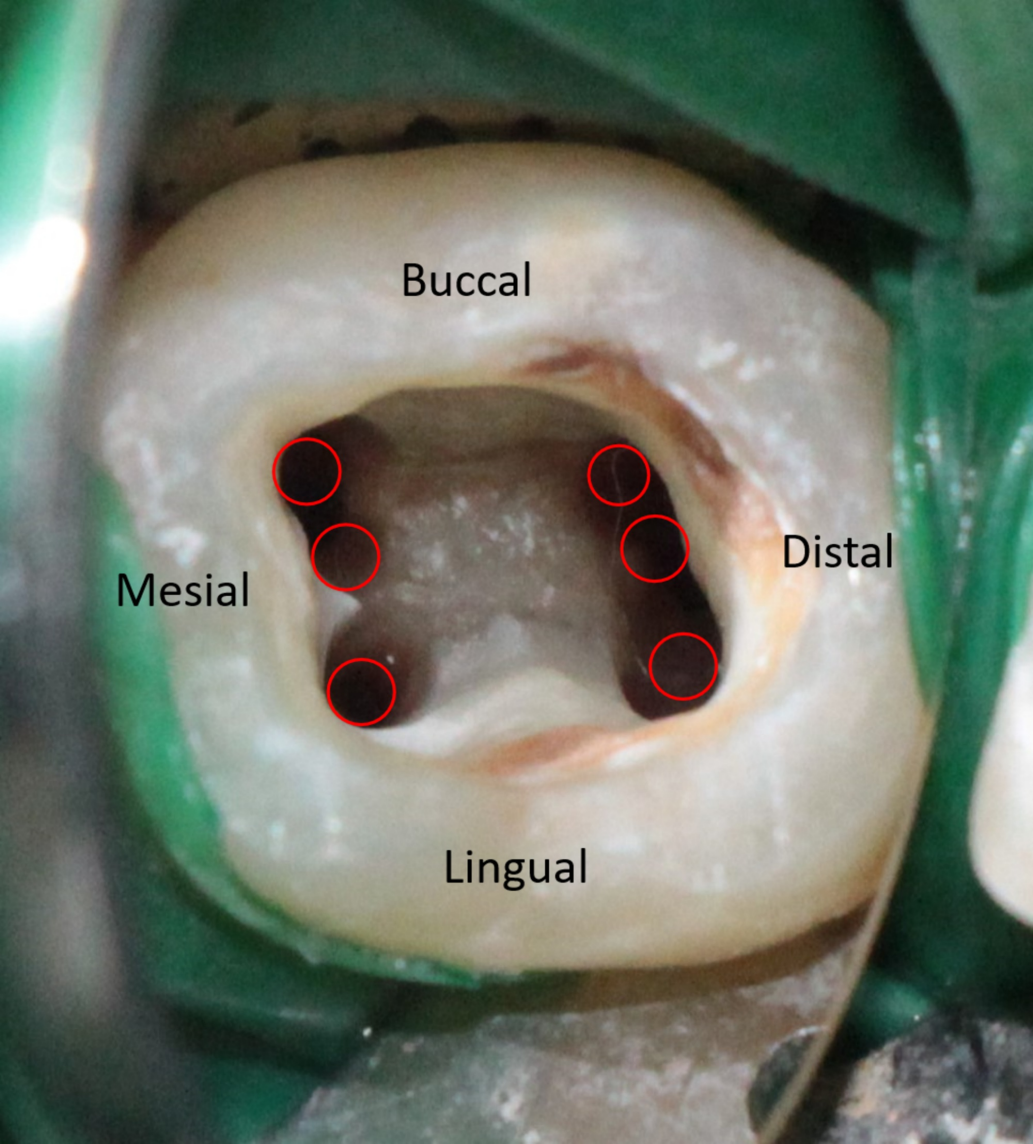
Access opening showing six distinct canal orifices

Careful exploration using a DG16 Endodontic Explorer (Manipal Instruments, Manipal, Karnataka, India) was done along the groove connecting mesiobuccal and mesiolingual orifice, and a third mid-mesial canal was found. The distal canal was positioned a little buccally, indicating the possibility of an extra canal, and a dark line was observed between the distal canal orifice and the distolingual corner of the pulp chamber floor. The conventional access was modified by extending the access cavity distolingually and a second distolingual canal orifice was detected. Exploration was carried out along the groove extending between distolingual and distobuccal canals, and an additional distobuccal canal was found. Thus, the pulpal floor revealed six distinct root canal orifices (Figure 2): three were detected mesially (mesiobuccal, middle mesial, and mesiolingual) and three distally.

**Figure 3:**
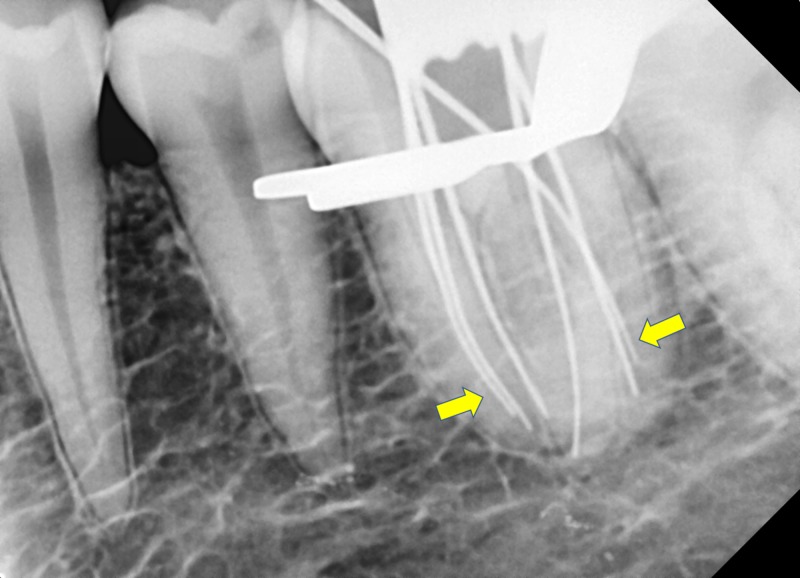
Working length radiograph showing six canals and extra distolingual root

After canal identification and negotiation, coronal enlargement was done with the help of Ni-Ti ProTaper gold orifice shaper (SX; Dentsply, Maillefer, Ballaigues, Switzerland) to improve the straight-line access of canals. The working length was then determined by electronic apex locator (Root Zx II, J Morita, Japan) and was verified with a radiograph (Figure 3). Interpretation of working length radiograph confirmed the presence of an additional distolingual root. Of the six distinct root canal orifices detected, three were detected mesially (mesiobuccal, middle mesial, and mesiolingual), indicating the Type XV (3-2) Sert and Bayirli canal configuration, and two canals were present in distobuccal root (distobuccal 1 and distobuccal 2) with Type II Vertucci canal configuration, and one independent canal in distolingual root.

**Figure 4:**
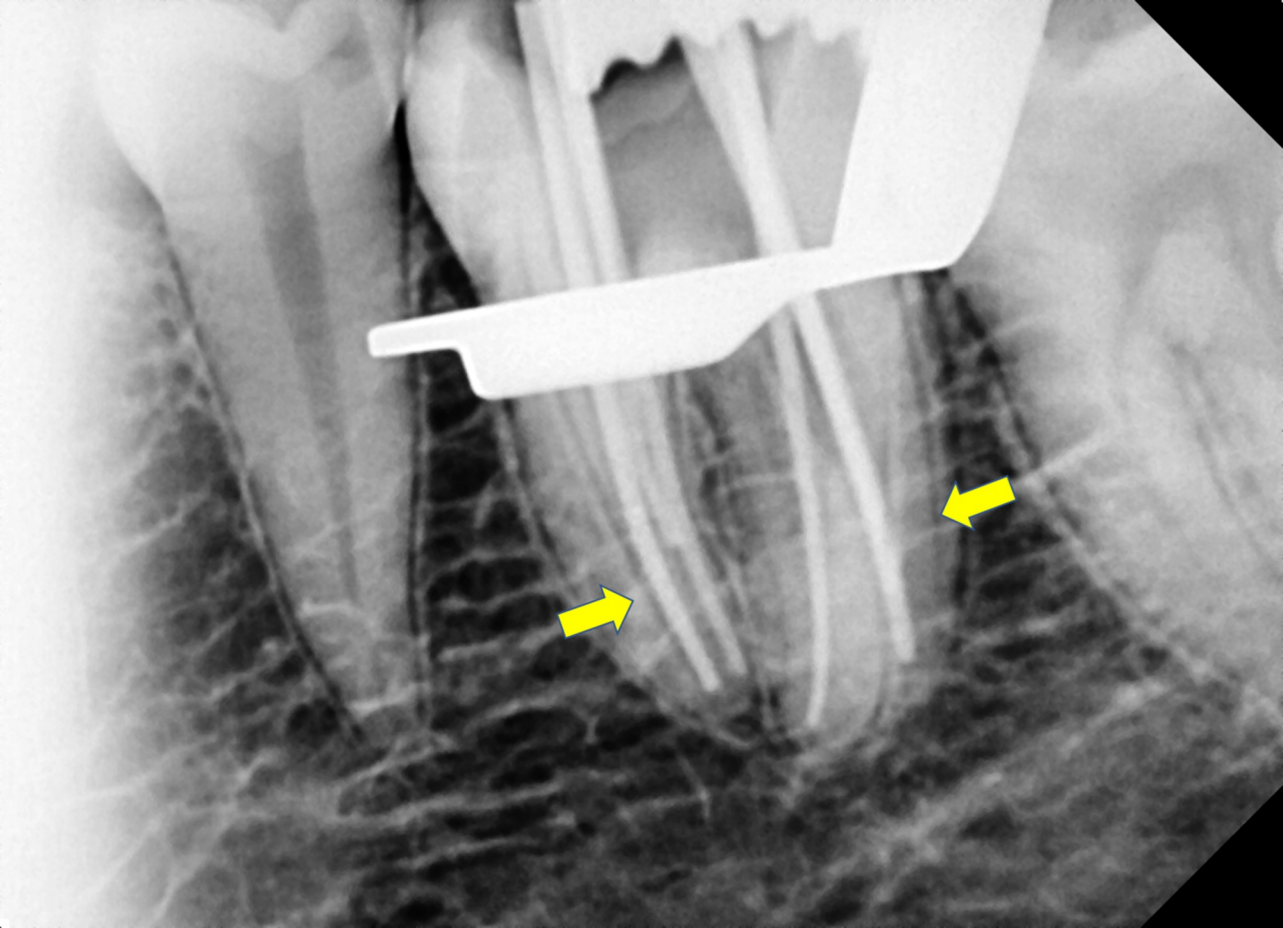
Master cone radiograph

**Figure 5:**
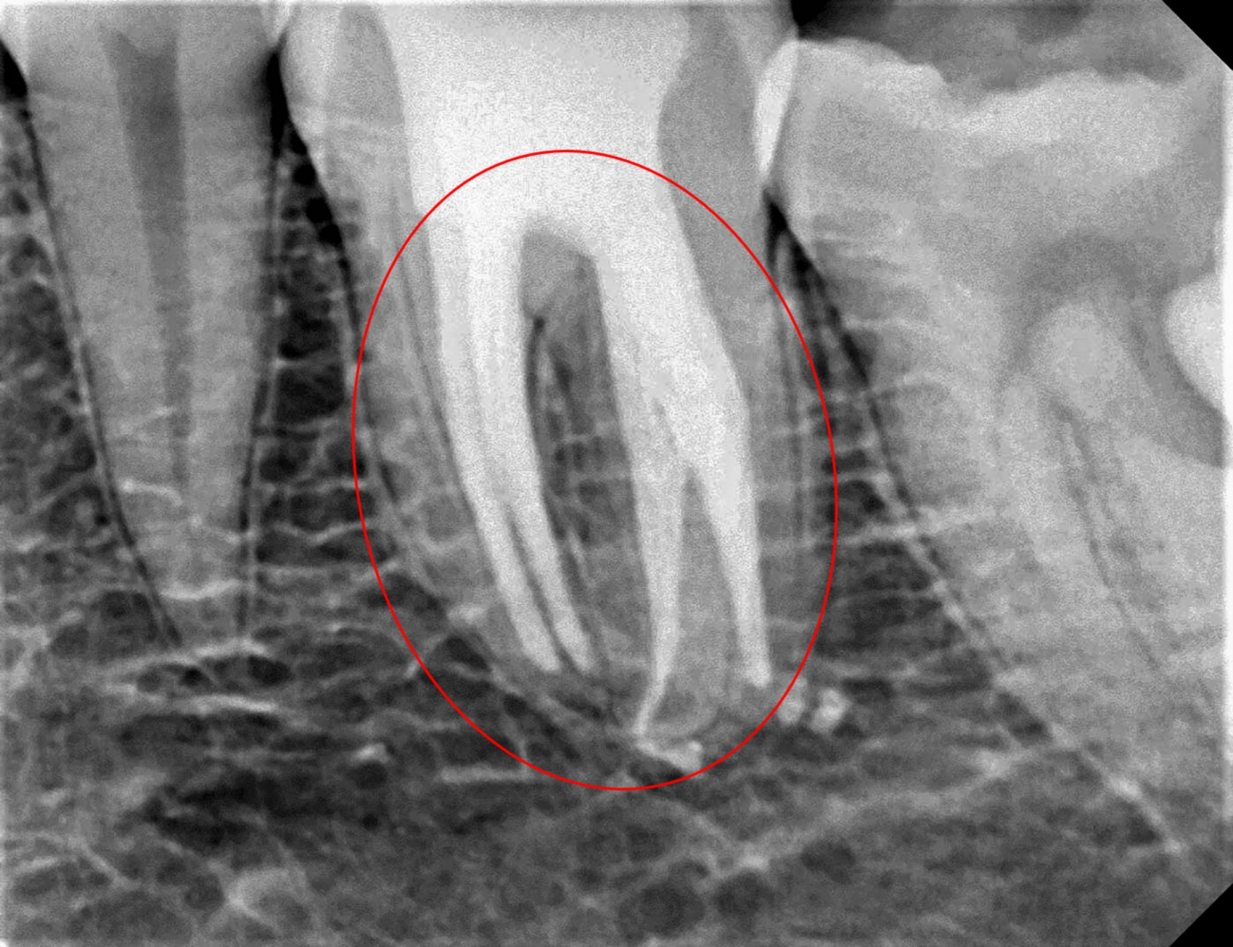
Post-obturation radiograph

Following working length determination, cleaning and shaping were done using Protaper Ni-Ti gold files using the crown down technique. All the instrumentation was performed with copious irrigation using 5.25% sodium hypochlorite and 17% ethylenediaminetetraacetic acid, alternatively. Irrigants were activated by sonic system (MM1500 Sonic Air, Micro Mega International, Besancon, France). Calcium hydroxide intracanal medicament (Vitapex, Neo-Dental International, Federal Way, WA) was placed in root canals, and access cavity was restored with temporary cement. At the second visit, after one week, calcium hydroxide medicament was removed, and a final rinse was done using sodium hypochlorite. Root canals were dried with absorbent points, and master cone was selected and positioned in respective canals, and radiograph was evaluated (Figure 4). Canals were then obturated with warm vertical compaction technique, and calcium hydroxide-based sealer (Sealapex, Kerr Dental, Orange, CA) was used. Post-obturation X-ray was evaluated (Figure 5), and at the six-month follow-up visit the patient was asymptomatic.

## Discussion

Various authors have reported the existence of mandibular first molar with additional root and extra canals [4,5]. Although preoperative radiograph has a key role in endodontic treatment, a minimum of two diagnostic periapical radiographs should be taken at different angulation for careful evaluation of the root canal morphology and to avoid chances of missing extra canals [6]. Krasner and Rankow proposed specific laws: the law of symmetry, the law of color change, and the laws of orifice location to locate canal orifices in an efficient manner [7]. Proper knowledge of root canal anatomy and its variations combined with use of various diagnostic aids such as spiral computed tomography, cone beam computed tomography, magnification devices, microscopes, performing sodium hypochlorite test will facilitate the identification of additional root and root canals.

The uniqueness of this case is the presence of six distinct root canal orifices and canals with extra distolingual root (radix entomolaris) in mandibular first molar. The mesial roots had canals with Type XV (3-2) as per Sert and Bayirli canal configurations, that is, the middle mesial canal merged with the mesiobuccal canal just above the apical third and exited through a common foramen. The mesiolingual canal was independent type with separate orifices and apical portal of exits.

In the distal root, distal buccal 1 and distobuccal 2 canal joined in its apical third having a mutual exit. The distolingual root had a separate canal. The presence of two distal roots is unusual but does occur. This additional root that can usually be found distolingually was first mentioned in literature by Carabelli and was called “radix entomolaris” [8]. A literature search showed that the prevalence of radix entomolaris in the mandibular first molar was reported to be 5.3% in the Indian population, 13.3% in South Indian by Chourasia et al. and Chandra et al., respectively [9,10]. Access cavity modifications are necessary (from triangle to rectangle or rhomboidal) to locate the additional canals in both mesial and distal roots. It has been reported that among the three-rooted mandibular first molars studied by the authors, the distolingual orifice was located 2.93 mm lingual to the distobuccal orifice, and the access form should be modified to a trapezoidal shape to expose all orifices [11].

Barker et al. and Vertucci and Williams first demonstrated the existence of a third root canal, in the mesial root of mandibular molars [12,13]. Since then, several authors have reported the existence of middle mesial canals. It has been hypothesized that the connective pulp tissue is likely to be compressed by the accumulation of secondary dentin during the growth of the root, which can, at times, form vertical walls, creating three independent mesial root canals [14]. Studies reveal that the incidence of negotiable middle mesial canals was found to be higher in younger patients [15,16]. In a clinical evaluation of 100 mandibular first and second molars, Pomeranz et al. found that 12 molars had mid-mesial canals in their mesial roots and classified them into three morphologic types as follows: fin, confluent, and independent, with the most prevalent anatomy being “fin” type [16]. However, Nosrat et al. reported that among the mandibular molar studied by the authors, 46.7% of teeth anatomy was confluent type, while Karapinar-Kazandag et al. found that in all the teeth studied by the authors, mid-mesial canal anatomy showed “confluent” anatomy [15,17]. The extra-mesial canal orifice found between the two main mesial canals is generally hidden by a dentinal projection of the pulp chamber wall, and this layer of dentin is differentiated from the pulp chamber floor by its lighter color. The mean diameter of mid-mesial canal orifice is reported to be 0.16 mm, which is three times less than the diameter of the two main orifices [18]. Enlargement of the middle mesial canal is more limited than for either the mesiobuccal or mesiolingual canal to avoid lateral perforation because the middle mesial canal is usually found in the narrowest portion mesiodistally of this root [16]. In addition, variation in location of the mid-mesial canal with respect to main canals has been reported by various authors. Karapinar-Kazandag et al. reported a higher number of additional mesial canals located closer to the mesiolingual canal in their study [17]. In a study evaluating the frequency of middle mesial canals in a North Indian population, the authors found that among 258 mandibular first molars, in 67% of the cases the mid-mesial canal orifice was located in the middle of the mesiobuccal and mesiolingual canal orifices, 20% had the orifice closer to the mesiolingual canal, while the remaining 12% had the orifices located closer to the mesiobuccal canal [19]. Differences in observation by various authors may be attributed to difference in study methodology, population studied, and number of teeth included in the study.

The use of modern diagnostic tools can prevent an inadvertent search for extra canals that can lead to excessive removal of tooth structure and perforations. In this case report, use of magnification loupes aided in the proper detection of extra canals. In addition, proper treatment protocol was followed that led to success of endodontic treatment.

## Conclusions

A thorough understanding of root canal anatomy is the key to successful root canal therapy. It is mandatory that clinicians should have a full understanding of the canal configuration and be aware of anatomical variations. Judicious interpretation of the radiographs, definite access preparation, and proper exploration of the pulp chamber under magnification, followed by proper cleaning and shaping of canals, are the fundamental prerequisites for a favorable outcome.
